# Location of Retroperitoneal Lymph Node Metastases in Upper Tract Urothelial Carcinoma: Results from a Prospective Lymph Node Mapping Study

**DOI:** 10.1016/j.euros.2023.09.010

**Published:** 2023-09-27

**Authors:** Johannes Bobjer, Axel Gerdtsson, Johan Abrahamsson, Gediminas Baseckas, Mats Bergkvist, Mats Bläckberg, Johan Brändstedt, Georg Jancke, Oskar Hagberg, Petter Kollberg, Karl-Johan Lundström, Annica Löfgren, Martin Nyberg, Liselotte Rian Mårtensson, Ymir Saemundsson, Elin Ståhl, Anne Sörenby, Åsa Warnolf, Fredrik Liedberg

**Affiliations:** a Department of Translational Medicine, Lund University, Malmö, Sweden; b Department of Urology Skåne University Hospital, Malmö, Sweden; c Pelvic Cancer Medical Unit, Karolinska University Hospital, Stockholm, Sweden; d Department of Urology, Helsingborg County Hospital, Helsingborg, Sweden; e Department of Clinical and Experimental Medicine, Division of Urology, Linköping University, Linköping, Sweden; f Department of Surgical and Perioperative Sciences, Urology and Andrology, Umeå University, Umeå, Sweden; g Department of Urology, Östersund County Hospital, Östersund, Sweden; h Department of Urology, Trondheim University Hospital, Trondheim, Norway

**Keywords:** Urinary tract cancer, Lymph node dissection, Lymph node metastases

## Abstract

**Background:**

There is limited information on the distribution of retroperitoneal lymph node metastases (LNMs) in upper tract urothelial carcinoma (UTUC).

**Objective:**

To investigate the location of LNMs in UTUC of the renal pelvis or proximal ureter and short-term complications after radical nephroureterectomy (RNU) with lymph node dissection (LND).

**Design, setting, and participants:**

This was a prospective Nordic multicenter study (four university hospitals, two county hospitals). Patients with clinically suspected locally advanced UTUC (stage >T1) and/or clinical lymph node–positive (cN+) disease were invited to participate. Participants underwent RNU and fractionated retroperitoneal LND using predefined side-specific templates.

**Outcome measurements and statistical analysis:**

The location of LNMs in the LND specimen and retroperitoneal lymph node recurrences during follow-up was recorded. Postoperative complications within 90 d of surgery were ascertained from patient charts. Descriptive statistics were used.

**Results and limitations:**

LNMs were present in the LND specimen in 23/100 patients, and nine of 100 patients experienced a retroperitoneal recurrence. Distribution per side revealed LNMs in the LND specimen in 11/38 (29%) patients with right-sided tumors, for whom the anatomically larger, right-sided template was used, in comparison to 12/62 (19%) patients with left-sided tumors, for whom a more limited template was used. High-grade complications (Clavien grade ≥3) within 90 d of surgery were registered for 13/100 patients. The study is limited in size and not powered to assess survival estimates.

**Conclusions:**

The suggested templates that we prospectively applied for right-sided and left-sided LND in patients with advanced UTUC included the majority of LNMs. High-grade complications directly related to the LND part of the surgery were limited.

**Patient summary:**

This study describes the location of lymph node metastases in patients with cancer in the upper urinary tract who underwent surgery to remove the affected kidney and ureter. The results show that most metastases occur within the template maps for lymph node surgery that we investigated, and that this surgery can be performed with few severe complications.

## Take Home Message

We assessed a predetermined template for lymph node dissection for upper tract urothelial carcinoma in the renal pelvis or proximal ureter. High-grade surgical complications were uncommon, and the majority of lymph node metastases were located within the suggested template.

## 1 Introduction

Two out of three upper tract urothelial carcinomas (UTUCs) are located in the renal pelvis [1]. UTUCs of clinical stage ≥cT2 account for approximately 45% of all renal pelvic tumors [2], [3], while urothelial carcinomas in the bladder account for 25%. Even if almost half of patients with UTUC have locally advanced disease, evidence regarding the indications, extent, and possible curative potential for retroperitoneal lymphadenectomy (LND) in conjunction with radical nephroureterectomy (RNU) for UTUC stage ≥cT2 is lacking. In the wake of recently reported survival benefits with adjuvant systemic therapies for urothelial carcinoma after surgery [4], [5], the importance of adequate lymph node staging has further increased. In addition, retrospective series of patients with UTUC and lymph node metastasis (LNM) have reported long-term survival after surgery alone [6], with survival rates comparable to those in bladder cancer with LNM after radical cystectomy and LND without perioperative chemotherapy [7], [8].

Lymphatic drainage from the upper urinary tract is side-specific and differs between the renal pelvis and the proximal, middle, and distal ureter [9]. However, the optimal extent of retroperitoneal LND in patients with UTUC is yet to be defined. Current knowledge regarding the location of LNMs is based on retrospective data for 73 patients with lymph node–positive UTUC in the renal pelvis and upper ureter who underwent retroperitoneal LND, and one prospective lymph node mapping study [10], [11], [12], [13].

The very limited and mainly retrospective evidence available in the literature and an unmet clinical need [14] prompted us to conduct a prospective lymph node mapping study in a setting with modern imaging and surgery to provide evidence on the location of LNMs in UTUC and to assess short-term complications after surgery.

## 2 Patients and methods

### 2.1 Patient population

**Fig. 1:**
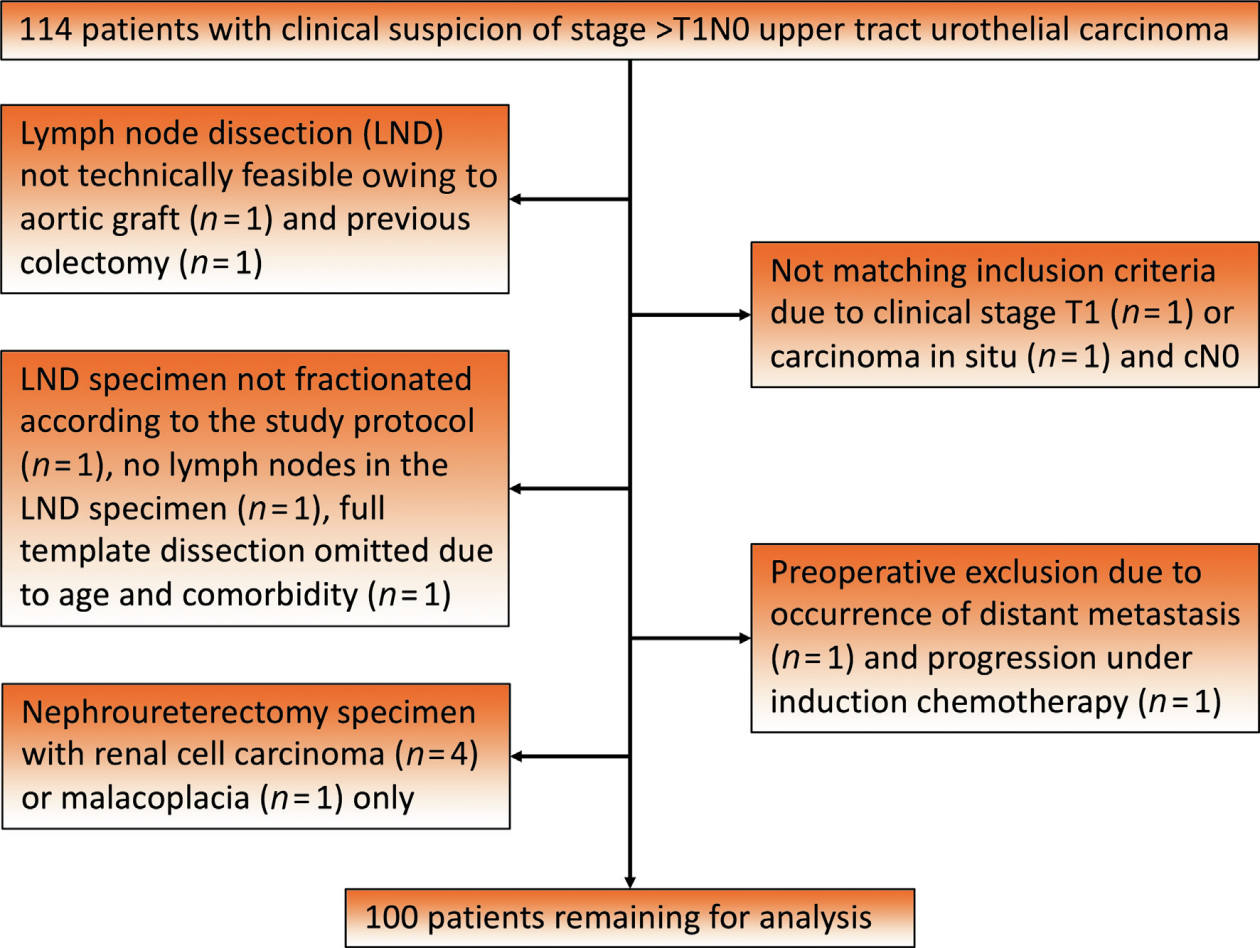
Consolidated Standards of Reporting Trials (CONSORT) diagram describing the study cohort.

Including seven feasibility cases performed in 2009–2012, a total of 114 patients with UTUC in the renal pelvis and/or proximal ureter (above the inferior mesenteric artery) and clinical suspicion of locally advanced tumor (≥cT2) or retroperitoneal lymph node involvement (cN+ disease) provided consent to participate in the study (ISRCTN83155790) up to 2022. The primary basis for clinical suspicion of locally advanced tumor stage was radiographic signs of parenchymal invasion and/or indirect signs such as hydronephrosis. The patients were treated at four university hospitals and two county hospitals in Norway and Sweden (Supplementary material). Chemotherapy (neoadjuvant or induction therapy for node-positive disease) was allowed before surgery. Exclusion criteria were age <18 yr and clinical tumor stage <cT2. In two of the 114 patients, previous abdominal surgery for inflammatory bowel disease with colectomy (*n* = 1) and an aortobifemoral graft for an aortic aneurysm (*n* = 1) meant that LND was not technically feasible. Before surgery, one patient developed distant metastases and one experienced disease progression on induction chemotherapy, and thus did not undergo RNU. Another two patients with clinical stage cT1 and carcinoma in situ only were excluded as they did not meet the inclusion criteria. In one patient the LND specimen was submitted en bloc, in one patient the specimen did not contain any lymph nodes, and in one patient the surgeon only performed a limited LND, so these patients were also excluded. Finally, five patients without UTUC in the RNU specimen were excluded (four renal cell carcinomas and one with malacoplacia). Thus, 100 patients remained for analyses (Fig. 1).

### 2.2 Diagnostic and staging procedures

Preoperative computed tomography (CT) or magnetic resonance urography was mandatory. Screening for metastases could be performed via thoracic CT or fluorodeoxyglucose (FDG) positron emission tomography (PET)/CT, according to the investigator choice. For patients who received induction chemotherapy, repeat CT or FDG PET/CT was performed to evaluate the response after three treatment cycles. Voided and/or selective urine cytology and ureteroscopy with or without biopsies were not mandatory but were performed according to the hospital routine. TNM staging according to the 2017 scheme was applied [15]. In Malmö University Hospital and Helsingborg County Hospital, a summary assessment by a regional multidisciplinary tumor board suggested invasive disease with clinical tumor stage >cT1N0 [16].

### 2.3 Intervention

**Fig. 2:**
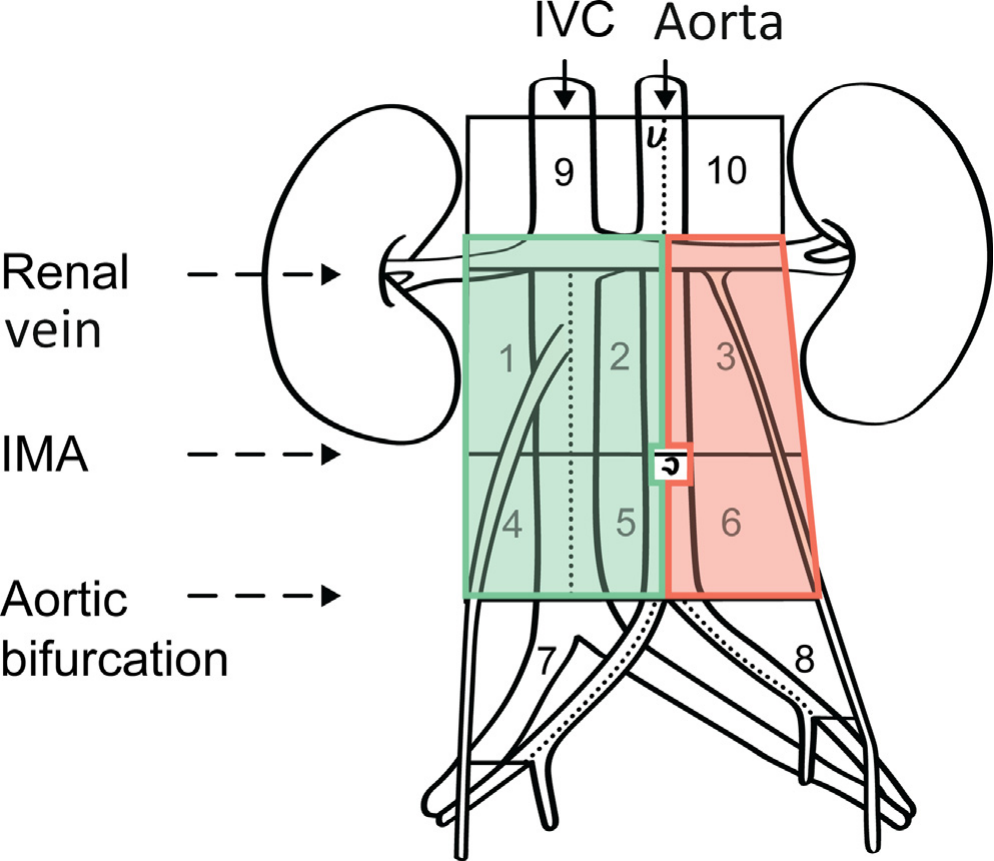
Lymphadenectomy was performed in four areas according to the green template (areas 1, 2, 4, and 5) for right-sided tumors, and in two areas according to the red template (areas 3 and 6) for left-sided tumors. IVC = inferior vena cava; IMA = inferior mesenteric artery.

Patients were selected for retroperitoneal LND using a right- or left-sided template for right- or left-sided UTUC, respectively. The template boundaries were defined using a similar retroperitoneal grid described for testicular cancer [17], except that the upper limit for areas 1, 2, and 3 was adjusted cranially to include the renal hilar nodes (Fig. 2). Thus, areas 1 and 4 include paracaval, precaval, and retrocaval nodal tissue around the vena cava, whereas areas 2 and 5 include the midcaval to midaortic area, covering medially located preaortal and retroaortal and precaval and retrocaval nodes. Areas 3 and 6 include the para-aortal area from the midaortic border laterally to the left ureter. The right ureter constitutes the lateral border for areas 1 and 4. The inferior mesenteric artery is the border between areas 1, 2, and 3, and areas 4, 5, and 6. According to the preference of individual surgeons, additional suspicious nodes were excised in separate fractions according to the same template description for either macroscopically suspicious lesions or findings on preoperative imaging. Both open and robot-assisted surgical approaches were allowed, with no specifications regarding surgical incision type or patient positioning.

### 2.4 Outcome measures

The primary outcome measure was the location of pathologically verified LNMs in the surgical template. The study protocol also included the location of later suspected retroperitoneal recurrences as an outcome for analysis. Postoperative complications within 90 d of surgery, assessed according to the highest Clavien grade, was a secondary outcome measure. In addition, recurrences during follow-up (distant metastases, retroperitoneal LNM and/or tumors in the urothelium) and deaths due to urothelial cancer or any cause were recorded.

### 2.5 Statistical analysis

Descriptive statistics were used to characterize the study population and to define rates of LNM and retroperitoneal recurrences in the side-specific template areas (right vs left), preoperative systemic treatment (yes vs no), and cN disease (cN0 vs cN1–2). The Kaplan-Meier method was used to plot curves for recurrence-free survival (RFS) and disease-specific survival (DSS) for patients with (pN1–2) or without (pN0) LNMs in the pathological LND specimen. In these analyses, time from surgery to the last day of follow-up was used. The median follow-up was 1.0 yr (interquartile range [IQR] 0.4–2.7) for RFS and 2.2 yr (IQR 0.8–4.6) for DSS.

Statistical analysis was performed using R (R Foundation for Statistical Computing, Vienna, Austria) [18].

### 2.6 Ethics

All patients included in the study provided oral and written informed consent before surgery. The study was approved by the Research Ethics Board at Lund University, Malmö, Sweden (Dnr 2013/321; 2016/645; 2018/84).

## 3 Results

In total, 100 evaluable patients underwent RNU and LND up to July 2022. The median age at surgery was 71 yr (IQR 64–75). Of the 100 eligible patients, 34 were female. There were 38 right-sided and 62 left-sided tumors. Preoperative FDG PET/CT was performed in 53 patients, and 29 patients had clinical node-positive disease detected preoperatively via either FDG PET/CT or standard CT (Table 1). Before surgery, nine patients received three neoadjuvant courses of methotrexate, vinblastine, adriamycin, and cisplatin (MVAC), while 15 patients with node-positive disease received induction chemotherapy comprising up to six courses of either MVAC (*n* = 10) or gemcitabine-carboplatin (*n* = 5) [19] (Table 1).

**Table 1:** Patient, tumor, and treatment characteristics for the study population (*n* = 100)

Parameter	Result
Median age at surgery, yr (IQR)	71 (64–75)
Sex (*n*)	
Female	34
Male	66
ASA score (*n*)	
1	14
2	52
3	33
4	1
Bladder cancer (*n*)	
Previous	9
Synchronous	7
Tumor location (*n*)	
Renal pelvis	78
Proximal ureter	7
Both locations	15
Tumor side (*n*)	
Right side	38
Left side	62
Preoperative FDG PET/CT (*n*)	53
Median T_Dmax_, cm (IQR)	4 (3–5) a
Clinical tumor stage (*n*)	
cT1 (N+)	2
cT2	28
cT3	67
cT4	3
Clinical nodal stage (*n*)	
cN0	71
cN1	8
cN2	21
Preoperative chemotherapy (*n*)	
Neoadjuvant	
MVAC	9
Induction	
MVAC	10
GCa	5
Adjuvant systemic therapy (*n*)	
MVAC	3
GC	1
GCa	8
Nivolumab	2

ASA = American Society of Anesthesiologists; IQR = interquartile range; FDG = fluorodeoxyglucose; PET/CT = positron emission tomography/computed tomography; T_Dmax_ = maximum tumor diameter; MVAC = methotrexate, vinblastine, adriamycin, and cisplatin; GC = gemcitabine and cisplatin; GCa = gemcitabine and carboplatin.

a Data from radiology and/or pathology report; values missing for 16 patients.

**Fig. 3:**
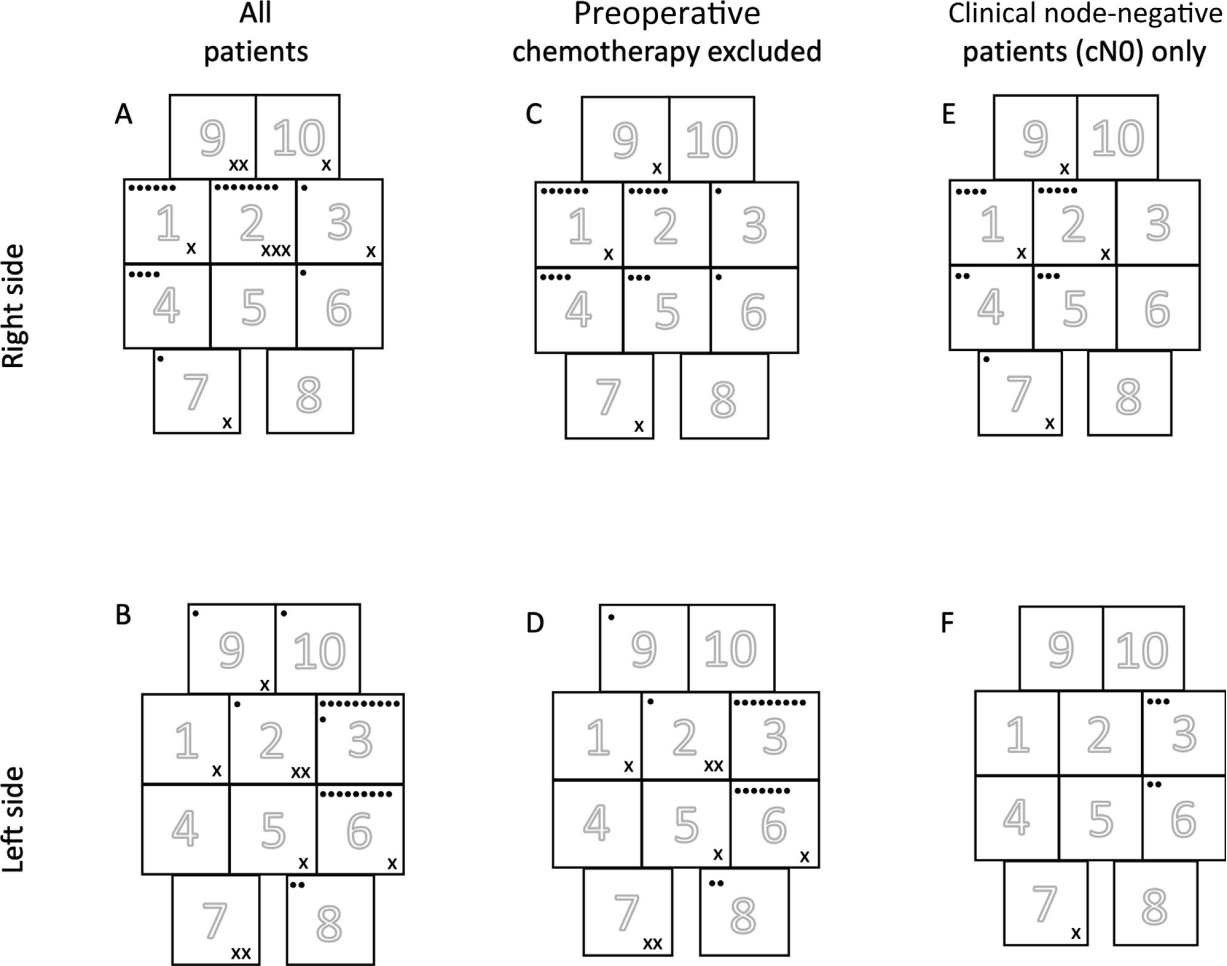
Location of histopathologically confirmed retroperitoneal lymph node metastases for patients with a right- or left-sided urothelial tumor in the pelvis or proximal ureter. A black dot represents one patient with a lymph node metastasis (pN1–2) in that area. An x represents one patient with a lymph node relapse (LNR) in that area during follow-up. Thus, an individual patient with disease in multiple template areas can be represented more than once. The corresponding proportions per patient with pN1–2 disease and LNR were as follows: (A) pN1–2: 11/38; LNR: 5/38; (B) pN1–2: 12/62; LNR: 4/62; (C) pN1–2: 7/28; LNR: 2/28; (D) pN1–2: 9/48; LNR: 3/48; (E) pN1–2: 7/30; LNR: 3/30; and (F) pN1–2: 3/41; LNR: 1/41.

LNMs were present in the LND specimen in 23/100 patients, of whom 19 (83%) had pN2 disease and seven (30%) had received preoperative chemotherapy (*n* = 5 induction therapy for node-positive disease, *n* = 2 neoadjuvant therapy without radiological signs of metastases; Table 2). One patient had multiple lymph nodes infiltrated by chronic lymphocytic leukemia at histopathology. LNMs were found in 11/38 (29%) right-sided tumors and 12/62 (19%) left-sided tumors. LNM locations are shown in Figure 3 for all patients and for subgroups who did not receive preoperative chemotherapy and with clinically node negative (cN0) disease. All the metastases extirpated were located within the field of the right-sided template (areas 1, 2, 4, and 5) in nine of 11 (82%) patients with right-sided UTUC, and within the field of the left sided template (areas 3 and 6) in eight of 12 (67%) patients with left-sided UTUC. In the subgroup with cN0 disease, the LNM rate was 86% (six of seven patients) for right-sided tumors and 100% (three of three patients) for left-sided tumors. In one patient with cN0 disease, intraoperative observation of an enlarged node in area 7 suspicious for metastasis prompted additional lymph node resection outside the defined right-sided template.

**Table 2:** Intraoperative characteristics and postoperative outcomes

Parameter	All patients (*n* = 100)	Right-sided tumors (*n* = 38)	Left-sided tumors (*n* = 62)
Surgical approach, *n* (%)			
Open	94	38 (100)	56 (90)
Robot-assisted	6	0	6 (10)
Median perioperative blood loss, ml (IQR)	300 (150–585)	300 (112–600)	300 (150–500) a
Median lymph node yield, *n* (IQR)	12 (8–22)	15 (8–25)	12 (7–19)
High-grade 90-d Clavien complications, *n* (%)			
Grade 3	6	3 (8)	3 (5)
Grade 4	3	0	3 (5)
Grade 5	4	1 (3)	3 (5)
pT stage, *n* (%)			
pTx	1	0	1 (2)
pT0	2	0	2 (3)
pTa	12	3 (8)	9 (15)
pTis	1	0	1 (2)
pT1	12	6 (16)	6 (10)
pT2	17	3 (8)	14 (23)
pT3	45	23 (61)	22 (35)
pT4	10	4 (11)	6 (10)
pN stage, *n* (%)			
pN0	77	27 (71)	50 (81)
pN1	4	2 (5)	2 (3)
pN2	19	9 (24)	10 (16)

IQR = interquartile range.

a One missing value.

During follow-up, five of 38 patients with a right-sided tumor and four of 62 with a left-sided tumor experienced a retroperitoneal recurrence at the denoted in Figure 3. Two of the patients with right-sided tumors and one patient with a left-sided tumor with retroperitoneal recurrences had negative findings for their LND specimens. Two of these three patients had tumor relapses outside the original template-based dissection, in template area 10 for one right-sided tumor and template area 7 for one left-sided tumor.

In 22/100 patients, histopathology revealed neither LNM in the LND specimen nor pathological tumor stage >pT1 in the RNU specimen. Seven of these patients had received preoperative chemotherapy (*n* = 4 neoadjuvant, *n* = 3 induction). Preoperative FDG PET-CT had been performed in 12 of these 22 patients, two of whom had suspicion of cN+ disease (both received induction chemotherapy).

### 3.1 Postoperative complications

Data on the incidence of Clavien grade ≥2 complications within 90 d of surgery are listed in Supplementary Table 1. In all, 13/100 of the patients suffered from high-grade complications (Clavien grade ≥3), and five patients underwent subsequent surgery for wound dehiscence (*n* = 1), small bowel leakage (*n* = 1), lymphorrhea (*n* = 1), and ileus (*n* = 2). Of the patients who received preoperative chemotherapy, two of 24 (8%) experienced high-grade complications, representing two of 15 (13%) who received induction chemotherapy and zero of nine who received neoadjuvant chemotherapy. All four deaths within 90 d of surgery (Clavien grade 5) were caused by disseminated urothelial carcinoma. Three of these four patients had cN+ and pN+ status and underwent surgery without any preoperative chemotherapy. The fourth death within 90 d of surgery occurred in a patient with cN0 and pN0 status with a locally advanced undifferentiated sarcomatoid carcinoma.

**Fig. 4:**
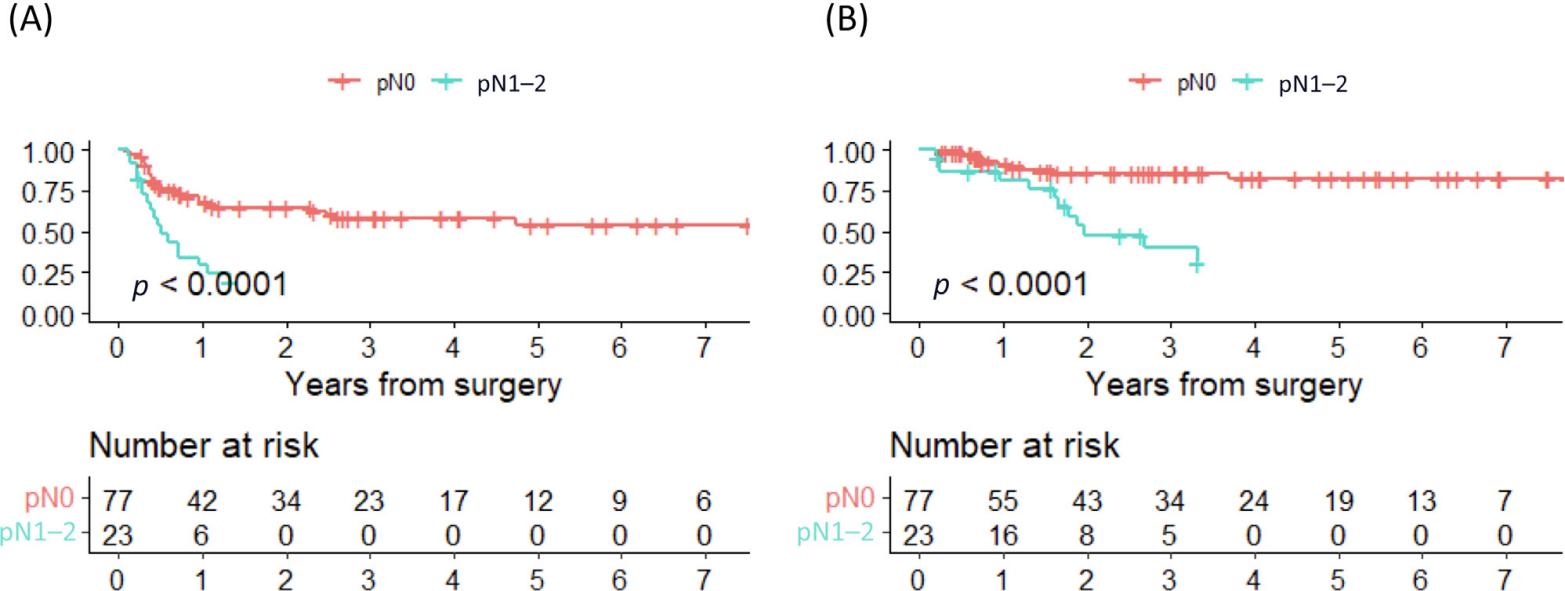
Kaplan-Meier curves for (A) recurrence-free survival and (B) disease-specific survival in patients with pN0 versus pN1–2 disease. Recurrence is defined as any of distant metastasis, retroperitoneal lymph node metastasis, or recurrent tumor in the urothelium. Disease-specific survival is defined as death due to urothelial carcinoma.

At the end of follow-up, ten of 77 (13%) patients with pN0 disease and 14/23 (61%) patients with pN1–2 had died from urothelial carcinoma. The rate of urothelial carcinoma recurrence was 26/77 (34%) forpN0 disease and 18/23 (78%) for pN1–2 disease. Kaplan-Meier curves for RFS and DSS for pN0 versus pN1–2 disease are shown in Figure 4. The nine patients with LNM in their LND specimens who were alive at the end of follow-up had all received systemic therapies in combination with surgery.

## 4 Discussion

Retroperitoneal lymphatic spread of UTUC in the renal pelvis and proximal ureter is complex and involved nine of the ten template areas investigated for both right-sided and left-sided tumors in our prospective study. Some 23% of our study patients harbored LNMs in the template areas dissected, with a larger anatomic region for right-sided tumors (template areas 1, 2, 4, and 5) than for left-sided tumors (template areas 3 and 6). High-grade complications directly related to the template-based LND occurred in two patients: one underwent subsequent surgery for persistent lymphorrhea and one was treated with percutaneous drainage for an infected lymphocele.

Supporting previous studies on lymph node mapping of LNM locations in patients with UTUC in the renal pelvis or proximal ureter [10], [11], [12], the current study corroborates lymphatic spread to a larger anatomic area in the retroperitoneum for right-sided tumors than for left-sided tumors. This was also true for the subset of patients who did not receive preoperative chemotherapy (Fig. 3C and 3D). It is also noteworthy that we only detected one cN0 patient with a crossover LNM (left to right) during follow-up (Fig. 3F), supporting the notion that the suggested template facilitates correct staging of locally advanced cN0 UTUC. In contrast to the only previous prospective study on lymph node mapping [13], we also included lymph node relapse during follow-up.

The 22 patients without >pT1 tumor or LNM in their LND specimen in our study reflect the difficulty in preoperative local staging for UTUC, although seven of these patients might have been downstaged as a result of preoperative chemotherapy. Identification of patients with stage >cT1 disease at risk of LNM, for whom template-based LND could be recommended, is a clinical dilemma. It has recently been suggested that local invasion into the renal parenchyma or periureteric tissue has high specificity (85%) for prediction of stage ≥pT3 disease, with potential to independently predict non–organ-confined disease in the RNU specimen [20]. Thus, such findings should trigger surgery with template-based LND in patients with UTUC. The limited sensitivity of CT in predicting locally advanced UTUC is illustrated by the fact that 39% of patients with stage ≤cT1 disease in one of the participating institutions had pathology confirmation of more advanced disease in the RNU specimen [21]. As CT has low sensitivity for LNM detection (25%) [22], FDG PET/CT should be considered as an additional modality for preoperative selection of patients with UTUC as candidates for template-based LND. The higher sensitivity of FDG PET/CT for LNM detection could increase the rationale for perform template-based LND in an individual patient considering the high specificity (84%) observed in a multicenter study [23]. Furthermore, it has been reported that FDG PET/CT can be used to monitor treatment response in patients treated with induction chemotherapy for LNM bladder cancer and to predict survival after consolidative surgery [24], findings that can probably be generalized to the cN+ UTUC setting. Therefore, when recruiting patients for the present study, we used FDG PET/CT more frequently in the later part of the trial, reflecting the unmet clinical need of preoperative prediction of lymph node–positive status. Another issue that warrants additional research is the higher proportion of patients with pN+ status on pathology for right-sided than for left-sided cN0 tumors (Fig. 3E and 3F) in our study.

The rate of high-grade postoperative complications in our cohort (13%) is higher than the 5% reported for two other series [13], [25]. It is possible that the low rate of robot-assisted surgery in our series (6%) may explain this difference in part, as it has been reported that the risk of complications is lower after robot-assisted RNU with regional LND than after open surgery (odds ratio 0.6, 95% confidence interval 0.4–0.8) [26]. Three of the four patients who died from urothelial carcinoma within 90 d of surgery did not undergo preoperative FDG PET/CT. The contemporary strategy involving LNM detection via preoperative FDG PET/CT might have triggered induction chemotherapy, with surgery only performed in cases showing a response to chemotherapy [27]. It is noteworthy that no increase in postoperative complications was observed for patients who had received preoperative chemotherapy. Moreover, as experience with template-based LND in patients with locoregionally advanced UTUC increases, the severity of complications after this procedure might decrease.

The limitations of the current study include the lack of review of radiological examinations used for clinical staging. Similarly, the lack of quality assessment of the individual LND procedures is a study limitation, although the median number of lymph nodes excised is 12, which is twice as many as in recent radiology staging trials.[22], [23]. In addition, allowing the inclusion of patients treated with preoperative chemotherapy might have altered the outcomes if ypN0 status is achieved in patients with cN+ disease and consequent downstaging of the primary tumor in the RNU specimen. However, current practice is to administer preoperative chemotherapy in this setting, especially considering the risk for cisplatin-eligible patients of becoming cisplatin-ineligible after RNU because of a decrease in renal function [28]. Moreover, the limited cohort size did not allow exploration of potential predictors of pN+ disease or lymph node relapse.

On the basis of the LNM distribution observed in the present study, our proposed areas for template-based LND can be considered for patients with >cT1 stage UTUC in the renal pelvis and upper ureter given that postoperative complications are carefully monitored in each unit applying these templates to ensure lack of additional LND-related morbidity. On the basis of the limited prospective data available, we plan to extend inclusion in the trial and would welcome participation by other centers.

## 5 Conclusions

The templates investigated for LND in UTUC of the renal pelvis and proximal ureter captured the majority of LNMs when using the proposed template borders, with a larger dissection area applied for right-sided tumors. High-grade complications as a consequence of the template-based LND were rare.

  ***Author contributions***: Johannes Bobjer had full access to all the data in the study and takes responsibility for the integrity of the data and the accuracy of the data analysis.

  *Study concept and design*: Liedberg.

*Acquisition of data*: Bobjer, Gerdtsson, Abrahamsson, Baseckas, Bergkvist, Bläckberg, Brändstedt, Jancke, Kollberg, Lundström, Nyberg, Rian Mårtensson, Saemundsson, Ståhl, Sörenby, Warnolf, Liedberg.

*Analysis and interpretation of data*: Bobjer, Gerdtsson, Hagberg, Liedberg.

*Drafting of the manuscript*: Bobjer, Gerdtsson, Liedberg.

*Critical revision of the manuscript for important intellectual content*: Abrahamsson, Baseckas, Bergkvist, Bläckberg, Brändstedt, Jancke, Hagberg, Kollberg, Lundström, Löfgren, Nyberg, Rian Mårtensson, Saemundsson, Ståhl, Sörenby, Warnolf.

*Statistical analysis*: Bobjer, Hagberg, Liedberg.

*Obtaining funding*: Liedberg.

*Administrative, technical, or material support*: Löfgren, Liedberg.

*Supervision*: Liedberg.

*Other*: None.

  ***Financial disclosures:*** Johannes Bobjer certifies that all conflicts of interest, including specific financial interests and relationships and affiliations relevant to the subject matter or materials discussed in the manuscript (eg, employment/affiliation, grants or funding, consultancies, honoraria, stock ownership or options, expert testimony, royalties, or patents filed, received, or pending), are the following: None.

  ***Funding/Support and role of the sponsor*:** This work was supported by the Swedish Cancer Society (CAN 2020/0709), Swedish Research Council (2021-00859), Lund Medical Faculty (ALF), Skåne University Hospital Research Funds, the Cancer Research Fund at Malmö General Hospital, Nordic Cancer Union, the Skåne County Council Research and Development Foundation (REGSKANE-622351), the Gösta Jönsson Research Foundation, the Foundation of Urological Research (Ove and Carin Carlsson bladder cancer donation), and the Hillevi Fries Research Foundation. The funding bodies had no direct role in the study.

## References

[1] Holmang S., Johansson S.L. (2006). Bilateral metachronous ureteral and renal pelvic carcinomas: incidence, clinical presentation, histopathology, treatment and outcome. J Urol.

[2] Olgac S., Mazumdar M., Dalbagni G., Reuter V.E. (2004). Urothelial carcinoma of the renal pelvis: a clinicopathologic study of 130 cases. Am J Surg Pathol.

[3] Hall M.C., Womack S., Sagalowsky A.I., Carmody T., Erickstad M.D., Roehrborn C.G. (1998). Prognostic factors, recurrence, and survival in transitional cell carcinoma of the upper urinary tract: a 30-year experience in 252 patients. Urology.

[4] Birtle A., Johnson M., Chester J. (2020). Adjuvant chemotherapy in upper tract urothelial carcinoma (the POUT trial): a phase 3, open-label, randomised controlled trial. Lancet.

[5] Bajorin D.F., Witjes J.A., Gschwend J.E. (2021). Adjuvant nivolumab versus placebo in muscle-invasive urothelial carcinoma. N Engl J Med.

[6] Lughezzani G., Jeldres C., Isbarn H. (2010). A critical appraisal of the value of lymph node dissection at nephroureterectomy for upper tract urothelial carcinoma. Urology.

[7] Stein J.P., Lieskovsky G., Cote R. (2001). Radical cystectomy in the treatment of invasive bladder cancer: long-term results in 1,054 patients. J Clin Oncol.

[8] Herr H.W., Donat S.M. (2001). Outcome of patients with grossly node positive bladder cancer after pelvic lymph node dissection and radical cystectomy. J Urol.

[9] Goltzman M.E., Gogoj A., Ristau B.T. (2019). The role of lymphadenectomy at the time of radical nephroureterectomy for upper tract urothelial carcinoma. Transl Androl Urol.

[10] Komatsu H., Tanabe N., Kubodera S., Maezawa H., Ueno A. (1997). The role of lymphadenectomy in the treatment of transitional cell carcinoma of the upper urinary tract. J Urol.

[11] Kondo T., Hara I., Takagi T. (2017). Template-based lymphadenectomy reduces the risk of regional lymph node recurrence among patients with upper/middle ureteral cancer. Int J Clin Oncol.

[12] Matin S.F., Sfakianos J.P., Espiritu P.N., Coleman J.A., Spiess P.E. (2015). Patterns of lymphatic metastases in upper tract urothelial carcinoma and proposed dissection templates. J Urol.

[13] Kondo T., Hara I., Takagi T. (2014). Template-based lymphadenectomy in urothelial carcinoma of the renal pelvis: a prospective study. Int J Urol.

[14] Lange S., Calleris G., Matin S.F., Roupret M. (2023). Optimizing lymph node dissection at the time of nephroureterectomy for high-risk upper tract urothelial carcinoma. Eur Urol Focus.

[15] Brierley J.D., Gospodarowicz M.K., Wittekind C. (2017). TNM classification of malignant tumours.

[16] Liedberg F., Sorenby A. (2020). Re: Improving management of upper tract urothelial carcinoma. Eur Urol.

[17] Gerdtsson A., Thor A., Grenabo Bergdahl A. (2022). Unilateral or bilateral retroperitoneal lymph node dissection in nonseminoma patients with postchemotherapy residual tumour? Results from RETROP, a population-based mapping study by the Swedish Norwegian Testicular Cancer Group. Eur Urol Oncol.

[18] R Core Team. R: A language and environment for statistical computing. Vienna, Austria: R Foundation for Statistical Computing; 2020. https://www.R-project.org/.

[19] Liedberg F., Abrahamsson J., Bobjer J. (2022). Re: Keisuke Shigeta, Kazuhiro Matsumoto, Koichiro Ogihara, et al. Does neoadjuvant chemotherapy have therapeutic benefit for node-positive upper tract urothelial carcinoma? Results of a multi-center cohort study. Urol Oncol. In press. https://doi.org/10.1016/j.urolonc.2021.07.029: A plea for uniform terminology for patients with urothelial carcinoma treated with chemotherapy before consolidative surgery with curative intent: induction versus neoadjuvant chemotherapy. Eur Urol.

[20] Almas B., Overby S., Halvorsen O.J., Reisaeter L.A.R., Carlsen B., Beisland C. (2021). Preoperative predictors of pathological tumour stage and prognosis may be used when selecting candidates for intensified treatment in upper tract urothelial carcinoma. Scand J Urol.

[21] Liedberg F., Abrahamsson J., Bobjer J. (2022). Robot-assisted nephroureterectomy for upper tract urothelial carcinoma-feasibility and complications: a single center experience. Scand J Urol.

[22] Pallauf M., D’Andrea D., Konig F. (2023). Diagnostic accuracy of clinical lymph node staging for upper tract urothelial cancer patients: a multicenter, retrospective, observational study. J Urol.

[23] Voskuilen C.S., Schweitzer D., Jensen J.B. (2020). Diagnostic value of ^18^F-fluorodeoxyglucose positron emission tomography with computed tomography for lymph node staging in patients with upper tract urothelial carcinoma. Eur Urol Oncol.

[24] Abrahamsson J., Kollberg P., Almquist H. (2022). Complete metabolic response with [18F]fluorodeoxyglucose-positron emission tomography/computed tomography predicts survival following induction chemotherapy and radical cystectomy in clinically lymph node positive bladder cancer. BJU Int.

[25] Rao S.R., Correa J.J., Sexton W.J. (2012). Prospective clinical trial of the feasibility and safety of modified retroperitoneal lymph node dissection at time of nephroureterectomy for upper tract urothelial carcinoma. BJU Int.

[26] Pearce S.M., Pariser J.J., Patel S.G., Steinberg G.D., Shalhav A.L., Smith N.D. (2016). The effect of surgical approach on performance of lymphadenectomy and perioperative morbidity for radical nephroureterectomy. Urol Oncol.

[27] Chakiryan N., Martinez A., Gao L. (2019). Optimizing the sequence of chemotherapy for upper tract urothelial carcinoma with clinically positive regional lymph nodes. J Urol.

[28] Shigeta K., Matsumoto K., Ogihara K. (2022). Does neoadjuvant chemotherapy have therapeutic benefit for node-positive upper tract urothelial carcinoma? Results of a multi-center cohort study. Urol Oncol.

