# Supplementary material for: Location of Retroperitoneal Lymph Node Metastases in Upper Tract Urothelial Carcinoma: Results from a Prospective Lymph Node Mapping Study
Source: Eur Urol Open Sci. 2023 Sep 27;57:37–44. doi: 10.1016/j.euros.2023.09.010 (PMC10658412; doi:10.1016/j.euros.2023.09.010)
Supplement: Supplementary Data 1 [file mmc1.docx]

*Supplemental material*

Location of retroperitoneal lymph node metastases in upper tract urothelial carcinoma – results from a prospective lymph node mapping study

PATIENTS AND METHODS

The following hospitals included patients in the study (number of patients included per hospital in brackets):

Trondheim University Hospital (n=9), Karolinska University Hospital (n=5), Linköping University Hospital (n=6), Skåne University Hospital Malmö (n=64), Östersund County Hospital (n=3), and Helsingborg County Hospital (n=13).

RESULTS

| Clavien- grade | n | Description of highest postoperative Clavien-grade complication within 90 days postoperatively (n) |
| --- | --- | --- |
| 2 | 28 | -postoperative infection (15)  -pulmonary embolism (2)  -atrial fibrillation (2)  -cardiac ischemia without myocardial infarction (1)  -total parental nutrition or blood transfusion (2)  -unplanned readmission (3)  -metabolic acidosis (1)  -gastric tube insertion (1)  -postoperative pain (1) |
| 3 | 6 | -reoperation for wound dehiscence (1)  -reoperation due to small bowel leakage (1)  -reoperation for lymphorrhea (1)  -reoperation for ileus (2)  -cerebrovascular insult with thrombectomy (1) |
| 4 | 3 | -renal insufficiency with temporary dialysis postoperatively (1)  -intensive care unit care due to respiratory, adrenal failure, and infected lymphocele (1)  -intensive care unit care for 24 hours due to inotropic support (1) |
| 5 | 4 | -dead of disseminated urothelial carcinoma (4) |

**Supplemental Table 1.** Types of postoperative complications grade 2 and higher within 90 days of surgery.
